# TLR4 signaling in VTA dopaminergic neurons regulates impulsivity through tyrosine hydroxylase modulation

**DOI:** 10.1038/tp.2016.72

**Published:** 2016-05-17

**Authors:** L Aurelian, K T Warnock, I Balan, A Puche, H June

**Affiliations:** 1 Department of Pharmacology, University of Maryland School of Medicine, Baltimore, MD, USA; 2 Neuropsychopharmacology Laboratory, Department of Psychiatry and Behavioral Sciences, Howard University College of Medicine, Washington, DC, USA; 3 Department of Anatomy and Neurobiology, University of Maryland School of Medicine, Baltimore, MD, USA

## Abstract

Alcohol dependence is a complex disorder that initiates with episodes of excessive alcohol drinking known as binge drinking, and has a 50–60% risk contribution from inherited susceptibility genes. Cognitive impulsivity is a heritable trait that may set the stage for transition to alcohol dependence but its role in the ethanol-seeking behavior and the involved genes are still poorly understood. We have previously shown that alcohol-preferring P rats have innately elevated levels of a neuronal Toll-like receptor 4 (TLR4) signal in the ventral tegmental area (VTA) that controls the initiation of excessive alcohol drinking. Here we report that TLR4 is localized in dopaminergic (TH+) neurons and it upregulates the expression of tyrosine hydroxylase (TH) through a cAMP-dependent protein kinase (PKA)/cyclic AMP response element binding protein (CREB) signal. P rats have higher impulsivity than wild-type (WT) rats and VTA infusion of a non-replicating Herpes simplex virus (HSV) vector for TLR4-specific small interfering RNA (siRNA; pHSVsiTLR4) inhibits both impulsivity and TLR4/TH expression. A scrambled siRNA vector does not affect gene expression or impulsivity. The data suggest that TLR4 signaling in VTA dopaminergic neurons controls impulsivity related to the regulation of TH expression, likely contributing to the initiation of alcohol drinking and its transition to alcohol dependence.

## Introduction

Alcoholism is a complex disorder that initiates with episodes of excessive alcohol drinking known as binge drinking (blood alcohol level ⩾0.08 g% in a 2-h period),^1^ and has a 50–60% risk contribution from inherited susceptibility genes.^2^ Neuronal functions that mediate pleasurable effects set the conditions for reward craving and the recruitment of mechanisms, which favor the transition to a relapsing course of sustained heavy drinking (alcohol dependence).^3^ Of particular interest is cognitive impulsivity, a heritable trait that correlates with addiction to virtually all drugs of abuse^4, 5^ and is believed to represent the ethanol-seeking behavior, which precedes steady alcohol consumption.^6, 7^ However, while alcohol-dependent individuals exhibit consistent findings of impulsivity-related deficits,^8, 9^ it is unclear whether these are specific to a small fraction of individuals who later become alcohol dependent and the involved genes are still poorly understood.

Neuroimmune signaling that includes the innate immunity receptor Toll-like receptor 4 (TLR4) was associated with a lifetime of alcohol consumption.^10, 11^ However, the potential contribution of genetic alterations to the initiation of excessive alcohol drinking, if any, is still poorly understood. We have shown that a neuronal TLR4 signal, which includes the downstream chemokine monocyte chemotactic protein (MCP-1, also known as CCL2) functions in the central nucleus of the amygdala and the ventral tegmental area (VTA) to control the initiation of alcohol drinking by alcohol-preferring P rats. The signal is sustained during alcohol drinking by increased expression of corticotropin-releasing factor and its feedback regulation of TLR4 expression, likely contributing to the transition to alcohol dependence.^12, 13^ Following on these findings and the observation that TLR4 contributes to the addiction-related reward system activity,^14^ the current studies considered the possibility that TLR4 controls the initiation of alcohol drinking through its effect on impulsivity.^6, 7^ They focus on the VTA, because it is a key player in the brain’s reward system and its dysregulation has long been implicated in cognitive behaviors that include addiction.^15, 16^ We report that the levels of TLR4 and tyrosine hydroxylase (TH) are higher in alcohol preferring P rats than wild-type (WT) rats. TLR4 localizes in dopaminergic (TH+) neurons and it induces TH expression through a cAMP-dependent protein kinase (PKA)/cyclic AMP response element binding protein (CREB) signal. The P rats have higher impulsivity than WT rats, and both impulsivity and TLR4/TH expression are inhibited by VTA infusion of a non-replicating Herpes simplex virus (HSV) vector (amplicon) for TLR4-specific small interfering RNA (siRNA; pHSVsiTLR4). Collectively, the data indicate that TLR4 signals through TH in VTA dopaminergic neurons to control impulsivity, potentially related to the initiation of alcohol drinking.

## Materials and methods

### Animals

Male alcohol-preferring (P) rats (*N*=52; 3–4 months old; 250–550 g) were obtained from the Alcohol Research Center, Indiana University School of Medicine. Male adult Sprague Dawley (SD) rats (*N*=20; 3–4 months old; 250–550 g) were obtained from Harlan Laboratories. The animals were individually housed, maintained at an ambient temperature of 21 °C and a reverse 12-h light/dark cycle and provided with food and water, *ad libitum*. Training and experimental sessions were conducted between 0830 and 1730 h. The treatment of all the subjects was approved by the IACUC of the Howard University College of Medicine and all the procedures were conducted in strict adherence with the National Institutes of Health Guide for the Care and Use of Laboratory Animals.

### Cells, plasmids, antibodies and reagents

The SK-N-SH human neuroblastoma cells (American Type Culture Collection, Manassas, VA, USA) were grown in RPMI 1640 medium with 2 mm l-glutamine (Gibco, Gaithersburg, MD, USA) and 10% fetal bovine serum (Gemini, West Sacramento, CA, USA). The TLR4^FLAG^ plasmid (#42646) is a gift from Scott Friedman and was obtained from Addgene (Cambridge, MA, USA). To establish stably transfected SK-N-SH cells, 50–80% confluent cultures (24 h post seeding) were transfected with TLR4^FLAG^ plasmid using FuGENE 6 Transfection Reagent (Promega, Madison, WI, USA). The TLR4^FLAG^ plasmid (20 μg) was incubated (15 min, room temperature (RT)) with FuGENE 6 Transfection Reagent in antibiotic-free medium before it was added to the cell cultures.

Antibodies to GAPDH (catalog (Cat.) #sc-47724), goat anti-TH (Cat. #sc-7847), and mouse and goat anti-TLR4 (Cat. #sc-293072, #sc-16240, respectively) were from Santa Cruz Biotechnology (Santa Cruz, CA, USA). The antibodies to phospho-CREB (pCREB; Ser-133; Cat. #9198) and phospho-PKA (pPKA; Thr197; Cat. #4781) and the PKA inhibitor H89 (Cat. #9844) were from Cell Signaling Technology (Danvers, MA, USA). Other antibodies were mouse anti-TH (EMD Millipore, Temecula, CA, USA; Cat. #MAB318), rabbit anti-TLR4 (Cat. #NBP1-78427, Novus Biologicals, Littleton, CO, USA), Alexa Fluor 488 goat anti-mouse IgG (H+L; Cat. #A11029) and Alexa Fluor 546 goat anti-rabbit or anti-mouse IgG (H+L; Cat. #A11035, #A11030, respectively; Life Technologies, Grand Island, NY, USA). Horseradish peroxidase-labeled secondary antibodies were anti-goat (Cat. #A24452, Life Technologies), anti-rabbit (Cat. #7074, Cell Signaling Technology) and anti-mouse IgG (Cat. #170-6516, Bio-Rad).

### Construction of TLR4 and scrambled amplicons

Amplicons are bacterial plasmids that are packaged into HSV type 1 (HSV-1) particles. They do not express viral proteins and are not toxic. We have previously described the construction and properties of the amplicon vectors for TLR4 (pHSVsiTLR4) and scrambled (pHSVsiNCC) siRNAs and showed that they do not cause loss of body weight or alter the general activity levels in P rats, do not induce cell death/apoptosis in intrastriatally infused animals and retain the HSV natural *in vivo* tropism for neurons.^12, 13^ This is further shown in Supplementary Data and it includes siRNA sequences and documentation of amplicon neuronal tropism (Supplementary Figure 1).

### Stereotaxic procedures

Amplicon delivery was as previously described.^12, 13^ The microinjection sites in the rat VTA extended from −5.0 mm posterior to bregma to −6.0 mm posterior to bregma, 0.6 mm lateral to the midline in both hemispheres and −8.2 mm into the brain from the surface of the skull.^17^ Because amplicons do not diffuse over long distances, we gave 13 small injections in each hemisphere spaced across the entire VTA. Each site received 200 nl of phosphate-buffered saline or amplicon (2.5 × 10^5^ transducing units) delivered with a calibrated pulled-glass micropipette (20-μm tip) connected to a Picospritzer II pneumatic pressure injection apparatus (Science Products, Hofheim, Germany). The injections were over 30 s followed by a 1- to 2-min pause for tissue recovery before insertion of the pipette at the next site. The Institutional Animal Care and Use Committee and Biosafety Committees of Howard University approved the procedures.

### Delay discounting (impulsivity)

The operant boxes consisted of a nosepoke light, two levers, a cue light above each lever, a house light and a 10-ml descending sipper tube for saccharin reinforcement (0.03% w/v).^6^ The boxes were controlled using MedPC IV software (St. Albans, VT, USA). The rats underwent five stages of behavioral shaping, with the final, fifth stage serving as 0-s delay testing,^6^ serving as a reinforcer magnitude discrimination task before introduction of any delay to the larger reward. Immediate reward amount started at 1 s of saccharin access, and was adjusted upwards and downwards by 0.1 s based on the rat’s choices. Forced trials of the non-selected reward followed two consecutive identical choices. Average adjusted amounts of the reward over the last 20 trials of the session served as the measure of adjusted amount. Lower adjusted amounts indicated increase impulsivity.^6^ If rats failed to complete 20 trials during stages 1–4 of shaping, they were excluded from testing. All the rats received 2-h water access in their home cages at the end of daily testing.

#### Phase 1

Following behavioral shaping, the P rats (*n*=10) and SD rats (*n*=10) were tested in the delay-discounting paradigm^6^ at 0, 1, 4, 8, 12, 16 and 20 s delays. Each delay was tested for two consecutive sessions and the 2-day data for each delay were averaged.

#### Phase 2

A separate group of P rats (*n*=32) were trained in the delay-discounting paradigm and tested at 0 and 12 s delays. These P rats were randomly separated into treatment groups, and bilaterally infused with pHSVsiTLR4 (*n*=14) or pHSVsiNCC (*n*=18). After 72 h of recovery, five rats infused with pHSVsiNCC and five rats infused with pHSVsiTLR4 were killed for immunoblotting evaluation. The remaining rats were tested in the delay-discounting paradigm at 4, 8, 12, 16, 20 and 25 s delays for two consecutive days at each delay. On days 19–22 of Phase 2, both treatment groups of rats were tested at 12 and 16 s delays to confirm the postviral effect. The 2-day data for each delay were averaged. Following the delay-discounting procedure on day 22 (16 days postviral vector administration), the rats were randomly selected from each treatment group (*n*=5 per treatment group) and killed for immunoblotting processing.

### Statistics

The data were analyzed by appropriate analyses of variance. Significant analyses of variance were followed by Newman–Keuls *post hoc* tests. The analyses were performed using the SigmaPlot 11.2 software program (Systat Software, San Jose, CA, USA).

### Immunoblotting

Immunoblotting was as previously described.^12, 13^ The SK-N-SH cells growing on T75 flasks (*n*=5 per treatment group) were lysed with radioimmunoprecipitation buffer (20 mm Tris-HCl (pH 7.4), 0.15 mm NaCl, 1% Nonidet P-40 (Sigma, St. Louis, MO, USA), 0.1% SDS (sodium dodecyl sulfate), 0.5% sodium deoxycholate) supplemented with protease and phosphatase inhibitor cocktails (Sigma).

The tissue brain micropunches (300-μm thick) obtained from naive P (*n*=5), SD (*n*=5) and P rats infused with pHSVsiTLR4 (*n*=5) or pHSVsiNCC (*n*=5) were lysed with CelLytic MT (dialyzable mild detergent, bicine and 150 mm NaCl; Sigma Aldrich, St. Louis, MO, USA) according to the manufacturer’s instructions. The total protein was determined by the bicinchoninic assay (BA; Pierce, Rockford, IL, USA). The proteins were resolved by SDS–polyacrylamide gel electrophoresis and transferred to polyvinylidene fluoride membranes. The blots were exposed to primary antibody overnight (4 °C), followed (1 h; RT) by horseradish peroxidase-labeled secondary antibodies. The detection was with the ECL kit reagents (Amersham Life Science, Pittsburgh, PA, USA) and quantification was by densitometric scanning with a Bio-Rad GS-700 imaging densitometer.

### Immunofluorescence

Immunofluorescent staining was as previously described.^13^ The SK-N-SH cells grown on poly-l-lysine (Sigma)-coated glass coverslips (*n*=5 per treatment group) were fixed with 4% paraformaldehyde (30 min; RT) and permeabilized (2 min; 4 °C) with 0.1% Triton X-100 in 0.1% sodium citrate buffer. They were exposed to primary antibody (diluted in 5% bovine serum albumin and 5% normal goat serum) overnight at 4 °C, washed in phosphate-buffered saline with 0.1% Tween 20 and exposed to fluorochrome-labeled secondary antibodies (1 h; RT).

Free-floating (30-μm thick) frozen sections were collected as described in Supplementary Materials and Methods, rinsed in phosphate-buffered saline, treated (95 °C, 10 min) with Retrievagen A (BD Pharmingen, San Jose, CA, USA), cooled (20 min, RT), and blocked with 5% goat serum (90 min, RT). The sections were obtained from five rats per treatment group. In each animal, four representative sections from 1:8 series throughout the VTA extending from −5.04 mm posterior to bregma to −6.00 mm posterior to bregma^17^ were exposed to primary antibodies (overnight, 4 °C) followed by the appropriate Alexa Fluor-labeled secondary antibodies (1 h, RT). Z-stack images (1 μm optical steps) were collected on an Olympus Fluoview FV5000 (Waltham, MA, USA) confocal microscope fitted with standard excitation and emission filters. The total number of TH+ cell bodies and cell bodies expressing both TH and TLR4 were counted in three randomly selected (× 40 magnified) images from each of the four studied sections and the % TH+ cells expressing TLR4 was calculated for each field. The results are expressed as mean±s.e. and analyzed by one-way analysis of variance followed by Newman–Keuls *post hoc* tests.

## Results

### The levels of TLR4 and TH are elevated in the VTA from P rats

**Figure 1:**
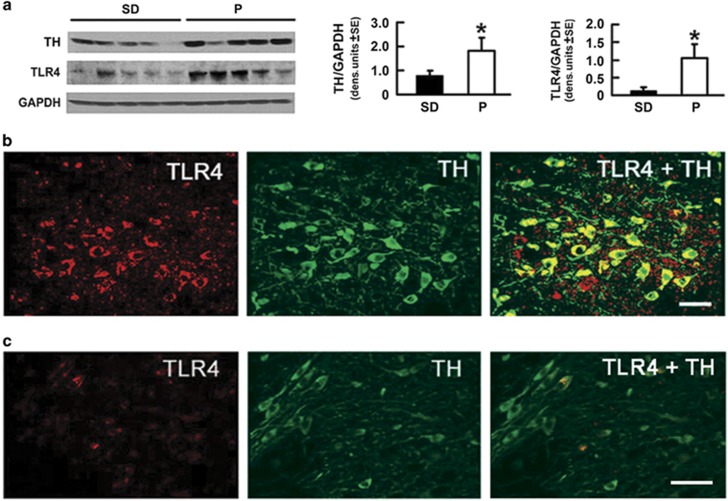
Toll-like receptor 4 (TLR4) and tyrosine hydroxylase (TH) are innately elevated and they co-localize in the ventral tegmental area (VTA) from alcohol-preferring (P) as compared with wild-type rats. (**a**) Protein extracts of micropunches collected from the VTA of Sprague Dawley (SD; *n*=5) and P (*n*=5) rats were immunoblotted with TH antibody were sequentially stripped and immunoblotted with antibodies to TLR4 and GAPDH used as a loading control. The results are GAPDH-normalized densitometric units±s.e.m. Each lane is a distinct animal. The TH and TLR4 levels are elevated in P as compared with SD rats. (*P<0.05 by analysis of variance). (**b** and **c**) Confocal microscopy and Z-stack imaging of double immunofluorescent staining with TLR4 (red) and TH (green) antibodies as described in the 'Materials and Methods' section is shown for the VTA of P (**b**) and SD (**c**) rats (*n*=5 per group). Merged images reveal numerous TH+ neurons expressing TLR4 in the VTA from P rats (**b**), but both the intensity and number of TLR4/TH+ positive cells (arrow) are significantly lower in the VTA from SD rats, as shown in **c**. Scale bars, 35 μm (**b**); 45 μm (**c**). Similar results were obtained in Wistar rats (*n*=5 per group; Supplementary Figure 2).

Following on previous findings that a neuronal TLR4 signal regulates binge alcohol drinking,^12, 13^ the current studies were designed to examine the relationship, if any, between TLR4 and innate impulsivity, the ethanol-seeking behavior that precedes steady alcohol consumption.^6, 7^ To avoid potential confounders resulting from genetic selection or rat strain (P rats were derived from Wistar rats), we first asked whether the levels of TLR4 are elevated in the VTA from alcohol-drinking P as compared with SD and Wistar rats, which drink similar levels of alcohol.^18, 19^ Because TH is the rate-limiting enzyme for the synthesis of catecholamine neurotransmitters, which function in impulsivity-associated behavior,^20, 21^ we considered the possibility that such elevation may be associated with TH modulation. The protein extracts from VTA micropunches were immunoblotted with TLR4 antibody and the stripped blots were re-probed with antibodies to TH followed by GAPDH, used as gel-loading control. The results were quantified by densitometric scanning and expressed as densitometric units normalized to GAPDH, as described in the 'Materials and Methods' section. The levels of both TLR4 and TH were significantly (*P*⩽0.05) higher in P than SD rats (Figure 1a), and a similar difference was seen when comparing P with Wistar rats (SD, Supplementary Figure 2A) or to non-alcohol-drinking NP rats.^12, 13^ The data indicate that P rats have elevated levels of TLR4 and TH in the VTA, independent of confounders resulting from genetic selection or strain.

### TLR4 is located in dopaminergic (TH+) neurons in the VTA

To examine whether the increased expression of TLR4 and TH in the VTA is associated with their cellular localization, we used double immunofluorescent staining with differentially labeled secondary antibodies. In P rats, TLR4 was strongly expressed (high intensity staining) in 61±5.2% of the TH+ neurons (Figure 1b), which are the major (55%) neuronal subpopulation in the VTA.^22^ Similar co-localization patterns were also seen in WT rats, but both the staining intensity and the % cells with TLR4/TH co-localized staining were significantly lower (12±5.5% and 10±4.9% for SD and Wistar rats, respectively; *P*⩽0.05; Figure 1c; Supplementary Figure 2B). We conclude that this is specific for TLR4, because α2 was also co-localized with TH (Supplementary Figure 4), but the % cells staining with antibodies to TH and α2 was similar in P and WT rats (86.2±4.0 and 87±3.8%, respectively). The data indicate that compared with WT rats, the P rats have a significant increase in the percentage of neurons that co-express TLR4 and TH. However TLR4 is also expressed in 83.3% of the GABAergic (GAD1+) neurons, which are a lower (38%) VTA neuronal subpopulation,^22^ and a similar co-localization was seen in both P and WT rats (Supplementary Figure 3).

### TLR4 upregulates TH expression through PKA/CREB phosphorylation

**Figure 2:**
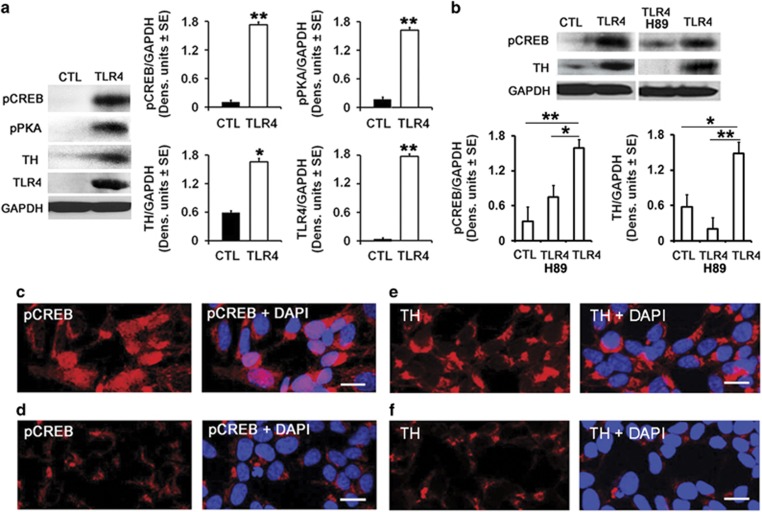
The tyrosine hydroxylase (TH), phospho protein kinase (pPKA) and phospho cyclic AMP response element binding protein (pCREB) expression is increased in Toll-like receptor 4 (TLR4)-transfected SK-N-SH cells. (**a**) Protein extracts from mock-(CTL) (*n*=5) and TLR4-transfected (*n*=5) SK-N-SH cells were immunoblotted with antibody to pCREB and the blots were sequentially stripped and re-probed with antibodies to pPKA, TH, TLR4 and GAPDH used as a gel-loading control. The results are expressed as GAPDH-normalized densitometric units±s.e.m. The levels of pCREB, pPKA and TH are significantly higher in SK-N-SH cells stably transfected with TLR4 than controls (*P<0.05; **P<0.01 by analysis of variance (ANOVA)). (**b**) pCREB and TH expression are reduced by treatment with the PKA inhibitor H89. Protein extracts collected from mock-(CTL) (*n*=5) and TLR4-transfected SK-NSH cells untreated (*n*=5) or treated (*n*=5) with the H89 (10 μm) were immunoblotted with antibody to pCREB and the blots were stripped and immunoblotted with antibodies to TH followed by GAPDH. The results were quantified by densitometic scanning and expressed as GAPDH-normalized densitometric units±s.e.m. The levels of pCREB and TH are significantly higher in TLR4- than mock-transfected cells and upregulation is inhibited by H89 treatment (*P<0.01; **P<0.01 by ANOVA). (**c**–**f**) Confocal microscopy of mock (**d** and **f**) and TLR4 (**c** and **e**)-transfected SK-N-SH cells stained with antibodies to pCREB (red) or TH (red) and DAPI (blue). Images are representative of findings from *n*=5 per treatment group. Numerous SK-N-SH cells stably transfected with TLR4 show pCREB expression in their nuclei (**c**) and the intensity and number of SK-N-SH cells expressing TH are significantly higher after TLR4 transfection (**e**). Scale bars, 20 μm (**c**–**f**).

Having seen that TLR4 localization in dopaminergic neurons is significantly higher in P than WT rats, we wanted to know whether the increased levels of TH seen in the P rats reflect the ability of TLR4 to upregulate TH expression. Two series of experiments were done. First, mock- and TLR4-transfected SK-N-SH cells were examined for TH expression by immunoblotting. The stripped blots were re-probed with antibody to the activated transcription factor CREB (pCREB) that is recognized by *cis*-response elements in the TH promoter, thereby inducing gene transcription (protein expression).^23, 24^ This was followed by immunoblotting with antibody to activated PKA (pPKA), which activates CREB through phosphorylation at Ser-133.^25, 26, 27^ Immunoblotting with GAPDH antibody was used as gel-loading control, and the results quantified by densitometric scanning are expressed as mean GAPDH-adjusted densitometric units±s.e. The levels of TH were significantly (*P*⩽0.05) higher in the TLR4- than mock-transfected cells, and this was accompanied by a significant (*P*⩽0.01) increase in the levels of both pPKA and pCREB (Figure 2a). The pCREB nuclear localization, which is indicative of activation,^27, 28^ was also seen in the TLR4- (Figure 2c) but not mock-transfected cells (Figure 2d) and the PKA-specific inhibitor H89 inhibited both the TH and pCREB upregulation (Figure 2b). Collectively, the data indicate that TLR4 upregulates TH expression through a pPKA/pCREB signal, consistent with findings implicating this signal in drug-induced TH control.^23^

**Figure 3:**
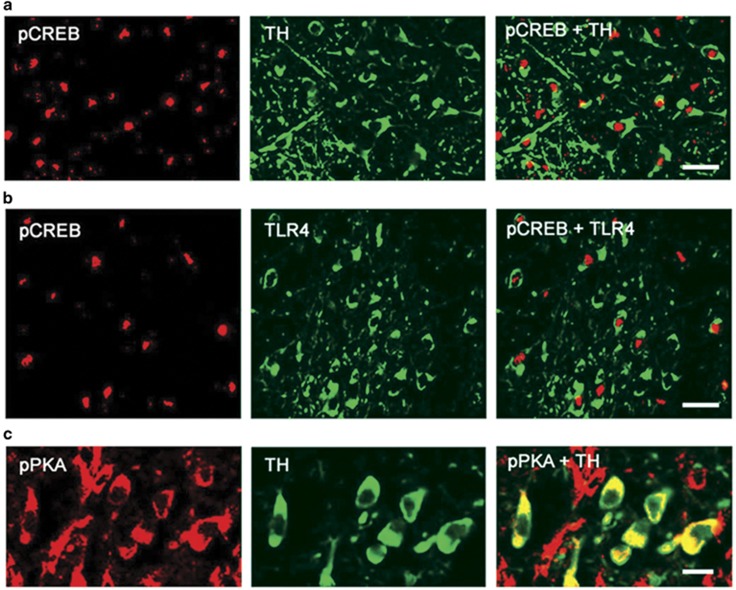
TH+ neurons co-expressing Toll-like receptor 4 (TLR4) in the ventral tegmental area (VTA) of alcohol-preferring (P) rats demonstrate positive immuno-reactivity for phospho cyclic AMP response element binding protein (pCREB) and phospho protein kinase (pPKA). Confocal microscopy and Z-stack imaging of double immunofluorescence staining for pCREB and tyrosine hydroxylase (TH; **a**), pCREB and TLR4 (**b**) and pPKA and TH (**c**) are shown for the VTA of P rats. Merged images for (**a**) pCREB (red) and TH (green), (**c**) pPKA (red) and TH (green) reveal TH-positive neurons co-express pCREB and pPKA in the VTA of P rats. TLR4-positive cells also co-express pCREB (**b**). Scale bars, 25 μm (**a** and **b**); 10 μm (**c**). Images are representative of findings from *n*=5 per treatment group.

The second series of experiments sought to confirm the co-expression of TH with the activated pPKA/pCREB functions in VTA sections that express TLR4. Duplicates of the serial VTA sections examined for TLR4 expression (Figure 1) were stained in double immunofluorescence with antibodies to TH and pCREB or pPKA. Both pCREB and pPKA co-localized with TH, with pCREB being primarily intranuclear (Figure 3a) and pPKA being primarily cytosolic (Figure 3c). Co-localization was also seen with TLR4, as shown for TLR4/pCREB in Figure 3b but pPKA/pCREB expression was barely detectable in SD rats, consistent with the minimal levels of TLR4 in these animals (Supplementary Figure 6). Collectively, the data indicate that TLR4 induces TH expression in VTA dopaminergic neurons from P rats through an activating PKA/pCREB signal.

### Innate impulsivity is higher in P vs SD rats

**Figure 4:**
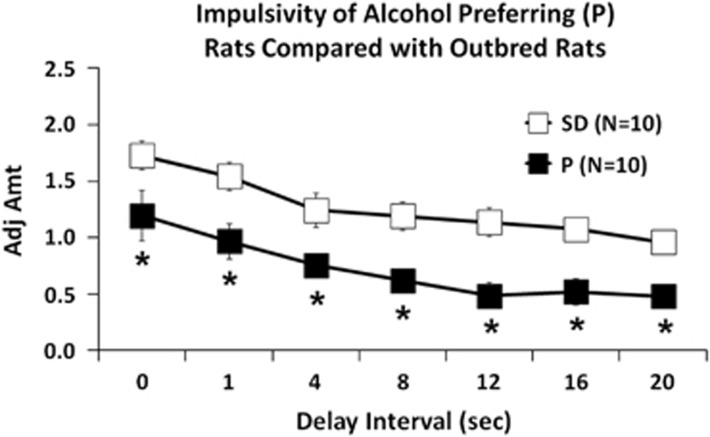
Innate impulsivity of P rats is greater than that of Sprague Dawley (SD) rats. Mean adjusted delay scores are significantly lower (impulsivity is elevated) in P than SD rats (*n*=10 each) for all the delays tested. *P⩽0.05 by analysis of variance.

Having seen that TLR4 upregulates the expression of TH, which functions in impulsivity-associated behavior,^20, 21^ we wanted to know whether the elevated levels of TLR4 seen in the P rats are associated with high levels of innate impulsivity. The data summarized in Figure 4 using the delayed discounting assay indicate that P rats have major impulsivity levels (lower adjusted amounts). These levels are significantly higher than the minimal levels seen in SD rats, with significant main effects of group (F_(__1,72)_=66.982, *P*<0.001) and delay (F_(6,72)_=16.206, *P*<0.001). *Post hoc* analyses confirmed the increased innate impulsivity of P rats as compared with SD rats for 0, 1, 4, 8, 12, 16 and 20 s delays (*P*⩽0.05).

### pHSVsiTLR4 infusion into the VTA decreases impulsivity in P rats, associated with decreased TH expression

**Figure 5:**
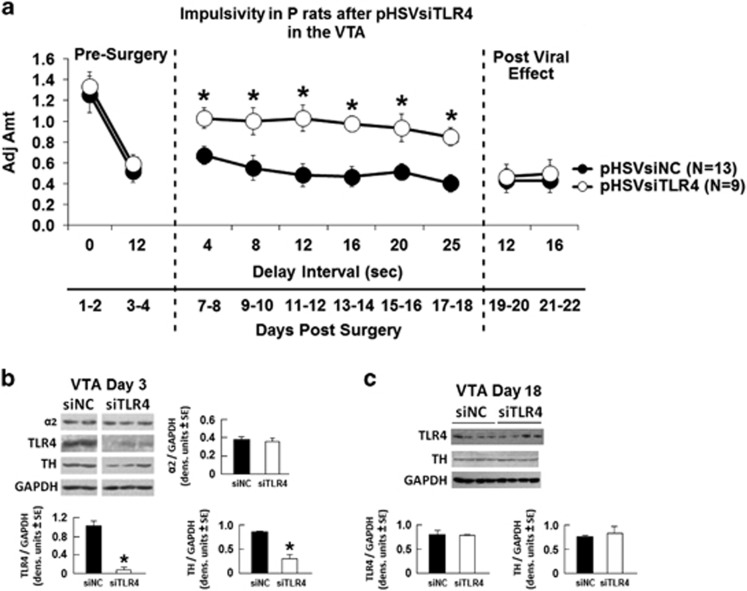
pHSVsiTLR4 infusion in the ventral tegmental area (VTA) inhibits impulsivity associated with Toll-like receptor 4 (TLR4) and tyrosine hydroxylase (TH) inhibition. (**a**) Mean adjusted delay scores are significantly increased (impulsivity is decreased) in P rats infused with pHSVsiTLR4 (*n*=9) relative to those of P rats injected with pHSVsiNCC (scrambled small interfering RNA (siRNA); *n*=13). Impulsivity is decreased on day 7 post surgery and returns to baseline levels on day 19. (*P*⩽0.05 by analysis of variance (ANOVA)). (**b**) pHSVsiTLR4 infusion in the VTA inhibits TLR4 and TH expression at 3 days after injection, but the levels are returned to baseline on day 18 post injection and expression is not inhibited by pHSVsiNCC (**c**). (*P*>0.05 by ANOVA). GABA_A_ α2 subunit levels were not affected by pHSVsiTLR4 injection.

To examine whether TLR4 and its downstream effector TH contribute to the increased impulsivity seen in the P rats, we used the pHSVsiTLR4 amplicon to inhibit TLR4 expression. A scrambled siRNA (pHSVsiNCC) amplicon served as control. The amplicons were previously described,^12, 13^ and the details of their construction, properties and neuronal localization are summarized in the Supplementary Materials and Methods and Supplementary Figure 1. pHSVsiTLR4 infusion into the VTA of P rats, significantly increased adjusted amounts (impulsivity was decreased) above those of P rats injected with pHSVsiNCC (Figure 5a). Significant main effects of treatment (F_(1,72)_=14.385, *P*=0.005), delay (F_(9,72)_=7.339, *P*<0.001) and treatment × delay interaction (F_(9,72)_=2.004, *P*=0.05) were seen. *Post hoc* tests confirmed an elevation of adjusted amount for 12 days across tested delay intervals of 4, 8, 12, 16, 20 and 25 s in pHSVsiTLR4-infused P rats compared with pHSVsiNCC-infused P rats (*P*⩽0.05). Significantly, the levels of TLR4 and TH expression reflected the effect of the pHSVsiTLR4 amplicon on impulsivity, being significantly (*P*⩽0.05) decreased at 3 days post injection (Figure 5b) and returning to the original pre-surgery values by day 16 after injection (Figure 5c), when the effect of the amplicon on impulsivity was no longer seen. The TLR4/TH inhibition is specific. It was not seen in the animals injected with pHSVsiNCC, and pHSVsiTLR4 had no effect on the expression of the GABA_A_ α2 subunit, which is upstream of TLR4 (ref. 12; *P*>0.05; Figure 5b). The duration of the pHSVsiTLR4 inhibitory effect on impulsivity is similar to that seen for binge drinking^12, 13^ and it reflects the duration of siRNA integrity/availability and the resulting posttranscriptional gene silencing.^12, 29^ Collectively, the data indicate that TLR4 controls impulsivity through a VTA signal that includes TH upregulation.

## Discussion

The salient feature of the data presented in this report is the finding that a TLR4 signal localized in the VTA dopaminergic (TH+) neurons regulates impulsivity in alcohol-preferring P rats through TH upregulation. The following comments seem pertinent with respect to these findings.

Alcoholism is a complex disorder that initiates with episodes of excessive alcohol drinking (binge drinking), which are associated with pleasurable effects that set the conditions for reward craving and favor transition to alcohol dependence.^3^ Inherited susceptibility genes were implicated in a lifetime of alcohol abuse but their role and function in the predisposition to initiate alcohol drinking is still poorly understood.^2^ Cognitive impulsivity is a heritable trait generally defined as a tendency to act without thinking. It correlates with addiction to virtually all drugs of abuse^4, 5^ and is believed to represent the ethanol-seeking behavior, which precedes steady alcohol consumption.^6, 7^ However, impulsivity is a multidimensional construct with a heterogeneous relationship to drug use^5, 6, 30^ and the genes involved in the predisposition to become alcohol-dependent and their function are still unclear. Using the five-choice serial reaction time task (5-CSRTT) to measure impulsivity, Pena-Oliver *et al.*^31^ have recently concluded that alcohol-preferring P rats are not intrinsically impulsive nor do they exhibit impulsivity after exposure to alcohol. Rather, their strong alcohol preference reflects increased goal-directed behavior to food incentives. By contrast, addictive behavior has been linked to paradigms of choice impulsivity (viz. delay-discounting tasks) in which impulsive individuals opt for immediate gratification that is detrimental in the long term over delayed benefits that have a more advantageous outcome in the long run.^32, 33^ Using this paradigm, high alcohol-preferring mice and rats showed steeper discounting (increased impulsivity) than low-preferring strains^6, 7, 34^ and alcohol-dependent individuals consistently displayed findings of impulsivity-related deficits.^8, 9, 35, 36, 37^

Dopaminergic neurons in the VTA are an integral part of the natural reward circuitry implicated in dependence produced by several drugs of abuse.^15, 20, 38^ Dopamine (DA) functions in impulsivity-associated reward, potentially regulating drug-seeking motivation.^39, 40^ Although DA may also be involved in preventing addiction,^41^ and lower or higher dopaminergic function was reported in distinct impulsivity-associated disorders,^21^ low D2/3 receptor availability and DA release in the striatum are considered neurobiological markers of increased impulsivity.^42, 43^ TH is the rate-limiting enzyme for the synthesis of impulsivity-regulating neurotransmitters,^20, 21^ and an established marker for the synthetic capacity of DA.^40, 42^ GABAergic response was also associated with cognitive impulsivity in adolescents and young adults^44, 45^ and genetic variation in the GABA_A_ α2 subunit was implicated in impulsivity and a lifetime of alcohol-related problems.^46^

Our studies follow on our previous findings that a neuronally localized TLR4 signal predisposes towards the initiation of alcohol drinking^12, 13^ and independent observations that TLRs expressed in neurons contribute to the regulation of cognition.^47, 48^ However, no study of which we are aware has previously demonstrated the role of TLR4 in impulsivity and the molecular mechanisms that might regulate such a function, are unknown. We addressed this question and focused on the possible contribution of TH as a downstream effector of TLR4, because of its well-established association with impulsivity-associated behavior,^20, 21, 39, 40^ and recent findings that associate TH polymorphisms with impulsivity-related traits, at least in dogs.^49^ The VTA was studied because it is implicated in cognitive behavior^15, 16^ and functions as a pivotal relay site within the reinforcement circuit involved in ethanol-motivated behaviors.^50^

We found that P rats had elevated levels of both TLR4 and TH in the VTA, when compared with SD and Wistar rats. The TLR4 signal was located in dopaminergic neurons, as evidenced by TLR4/TH co-localization in a significantly higher percentage of cells in P (61±5.2%) than WT rats (12±5.5% and 10±4.9% in SD and Wistar rats, respectively). This differential co-localization appears to be specific for TLR4, because the % cells with α2/TH co-localization was similar in P and WT rats (86.2±4.0 and 87±3.8%, respectively; Supplementary Figure 4). We conclude that TH functions as a downstream effector of the TLR4 signal, because (i) the levels of TH were significantly higher in TLR4- than mock-transfected SK-N-SH cells, (ii) TH upregulation in TLR4-, but not mock-transfected cells was accompanied by a significant increase in the levels of activated PKA/CREB and (iii) both the levels of pCREB and TH were significantly decreased in TLR4-transfected SK-N-SH cells treated with the PKA inhibitor H89 (Figure 2b), indicating that TLR4 upregulates TH expression through a PKA/CREB signal.

CREB is a transcription factor that is activated through phosphorylation by activated PKA (pPKA, Thr197) at Ser-133 (pCREB),^25, 26, 51^ and it translocates to the nucleus to control the transcription of genes, which contain cyclic AMP response elements (CRE) in their promoters.^27, 28^ Extensive studies of the human and rodent TH gene promoter have identified canonical and non-canonical CRE motifs that bind CREB and modulate expression as determined through transcription reporter and electromobility shift assays.^24, 52^ CRE variants were implicated in TH deficiency in individuals who lack mutations in the TH coding region, supporting the important role of CREB in TH expression.^53^ However, we recognize that cAMP regulates CREB also in a PKA-independent pathway, and we cannot exclude the possibilities that H89 may also have PKA-unrelated functions and PKA may directly phosphorylate TH (at Ser40).^54, 55, 56^ Moreover, in addition to transcription, complete spatial and temporal regulation of TH expression likely requires interactions between the promoter and distal enhancer or repressor *cis*-regulatory elements and posttranscriptional and posttranslational mechanisms that are still poorly understood.^56, 57^

Consistent with the conclusion that TLR4 regulates TH through a PKA/CREB signal, we found that both pPKA and pCREB are expressed in the VTA dopaminergic (TH+) neurons from P rats, where they co-localize with TLR4 (as shown for pCREB in Figure 3b). In these cells, pCREB is primarily intranuclear (activated), with relatively few cells showing TH/cytosolic pCREB co-localization (Supplementary Figure 5). pPKA/pCREB expression and co-localization with TH or TLR4 are barely detectable in SD rats (Supplementary Figure 6), supporting the interpretation that elevated TH expression in the VTA from P rats is through a TLR4-regulated PKA/CREB pathway. In this context, it seems particularly important to point out that (i) pCREB appears to have an important role in the addiction process^57, 58^ and (ii) the PKA/pCREB signal has been implicated in the molecular changes that underlie alcohol drinking and alcoholism.^59, 60, 61^ However, although our data document increased TLR4 expression in the VTA from alcohol-drinking P rats, the ligands responsible for its activation and PKA/pCREB/TH upregulation are still unclear. The bacterial endotoxin LPS (lipopolysaccharide) is the natural TLR4-activating ligand but other activating ligands were also described, including endogenous danger-associated molecular patterns released as a consequence of injury and inflammation, such as heat-shock proteins, extracellular matrix molecules (hyaluronan), HMGB1, oxidized low-density lipoprotein and oxidized phospholipids.^62, 63, 64^ These are unlikely to be involved in our system, because the PKA/pCREB/TH signal was not seen in WT animals, which are also likely to be LPS+, and all the groups were free of overt injury/inflammation. However, we do not exclude the possible involvement of recently identified agonists, such as saturated fatty acids, LPS mimetic ligands of natural origin, including polypeptides and neuron-derived IgG in dopaminergic neurons.^65, 66, 67^

Using the delay-discounting assay, we found that P rats had significantly increased levels of impulsivity than the SD rats, and these were blunted by VTA infusion of the pHSVsiTLR4 amplicon that specifically inhibited the expression of TLR4 together with its downstream target TH. Impulsivity was not altered by pHSVsiNCC that did not affect TLR4 and TH expression, and pHSVsiTLR4 did not alter the expression of α2, which is upstream of TLR4.^12^ Because pHSVsiTLR4 inhibits the volition to initiate alcohol drinking,^12, 13^ these data suggest that a VTA TLR4/TH signal may control the initiation of alcohol drinking through its ability to control impulsivity. However, we recognize that other unstudied variables may impact these findings, most notably TLR4-activating ligands, and we do not discount the potential contribution of the assay used to measure impulsivity. We also do not know whether and how α2 and MCP-1, both of which were implicated in drinking initiation^12, 13^ contribute to impulsivity and the role of PKA-mediated TH phosphorylation,^57^ pCREB mitochondrial localization^27^ and TLR4 expression in GABAergic (GAD1+) neurons, is still unknown. Also, the precise role of impulsivity in the vulnerability to engage in excessive alcohol drinking,^34^ particularly as it relates to specific brain sites and TLR4-activating ligands, remains to be addressed. Ongoing studies are designed to address these questions.

## Supplementary Material

Supplementary Informations
