# Supplementary material for: TLR4 signaling in VTA dopaminergic neurons regulates impulsivity through tyrosine hydroxylase modulation
Source: Transl Psychiatry. 2016 May 17;6(5):e815–. doi: 10.1038/tp.2016.72 (PMC5727490; doi:10.1038/tp.2016.72)
Supplement: Supplementary Informations [file tp201672x1.doc]

**SUPPLEMENTARY DATA**

Aurelian et al.

**SD MATERIALS AND METHODS**

**Cells, antibody and reagents**. Vero 2.2 cells that express the HSV gene ICP27 were cultured in DMEM-10% FBS and used as packaging cells for amplicon vector construction and rat pheochromocytoma (PC12) cells were grown in DMEM with 10% heat-inactivated horse serum and 5% FBS and used for amplicon vector titration, as previously described . The generation and specificity of the rabbit-derived GABAA α2 antibody was previously described; it recognizes amino acids 322-357 of the α2 protein (1). Antibodies to GAPDH (Cat.# sc-47724), goat anti-TH (Cat.#sc-7847), and mouse and goat anti-TLR4 (Cat.## sc-293072, sc-16240, respectively) were from Santa Cruz Biotechnology (Santa Cruz, CA). The antibodies to phospho-CREB (pCREB; Ser133) (Cat.# 9198) and phospho-PKA (pPKA; Thr197) (Cat.# 4781) were from Cell Signaling Technology (Danvers, MA). Other antibodies were GAD67 (GAD1) (LifeSpan BioSciences, Seattle, WA, Cat.# LS-B3027), mouse anti-TH (EMD Millipore, Temecula, CA, Cat# MAB318), rabbit anti-TLR4 (Cat.# NBP1-78427, Novus Biologicals, Littleton, CO), Alexa Fluor 488 goat anti-mouse IgG (H+L) (Cat.# A11029), Alexa Fluor 488 donkey anti-goat IgG (Cat.# A11055), and Alexa Fluor 546 goat anti-rabbit or anti-mouse IgG (H+L) (Cat.## A11035, A11030, respectively) (Life Technologies, Grand Island, NY). Horseradish peroxidase-labeled secondary antibodies were anti-goat (Cat.# A24452, Life Technologies) and anti-mouse IgG (Cat.# 170-6516, Bio-Rad).

**Collection of brain sections for immunofluorescent staining.** Rats were deeply anesthetized by intraperitoneal injection of sodium pentobarbital (Nembutal; 150 mg/kg, Abbott Laboratories, Abbott Park, IL, USA) and transcardially perfused with 0.9% saline followed by 4% paraformaldehyde (PFA) in 0.1 M phosphate buffer (PBS, pH 7.4). Brains were carefully dissected, post-fixed in 4% PFA (overnight, 4°C), incubated in 30% sucrose (48 h, 4°C) and mounted in OCT embedding compound. They were frozen at -20 to -40°C and coronally sectioned (30 μm) using a Leica CM3050 cryostat (Leica, Deerfield, IL, USA). The serial sections were kept in cryoprotectant (-20°C) until staining.

**Immunofluorescent staining**. Free-floating (30-μm thick) frozen sections were rinsed in PBS, treated (95°C, 10 min) with Retrievagen A (BD Pharmingen), cooled (20 min, RT), and blocked with 5% goat serum (90 min, RT). Sections were obtained from 5 rats/treatment group. In each animal, four representative sections from 1:8 series throughout the VTA extending from -5.04 mm posterior to bregma to -6.00 mm posterior to bregma were exposed to primary antibodies (overnight, 4°C) followed by the appropriate Alexa Fluor-labeled secondary antibodies (1h, RT). Z-stack images (1µm optical steps) were collected on an Olympus Fluoview FV5000 confocal microscope fitted with standard excitation and emission filters . Total number of TH+ or GAD1+ cell bodies and cell bodies expressing both TH and TLR4, TH and α2, and GAD1 and TLR4 were counted in three randomly selected (40x magnified) images from each of the 4 studied sections and the % TH+ cells expressing TLR4 or α2, and the % GAD1+ cells expressing TLR4 were calculated for each field. The results are expressed as mean ± SE and analyzed by one-way ANOVA followed by Newman-Keuls post-hoc tests.

**Stereotaxic Procedures**. Rats were anesthetized by intraperitoneal injection of nembutal (50 mg/kg) and positioned in a stereotaxic apparatus . The microinjection sites in the rat VTA extended from -5.0 mm posterior to bregma to -6.0 mm posterior to bregma, 0.6 mm lateral to the midline in both hemispheres, and -8.2 mm into the brain from the surface of the skull . Because amplicons do not diffuse over long distances, a single large injection would fail to cover the entire VTA and likely result in a pressure lesion. Accordingly, we gave 9 or 13 small injections in each hemisphere spaced across the entire VTA. Each injection site received 200 nL of PBS or amplicon (2.5 × 10^5^ TU) delivered with a calibrated pulled glass micropipette (approximately 20-μm tip) connected to a Picospritzer II pneumatic pressure injection apparatus (Science Products GmbH). Injections were over 30 s followed by a 1- to 2- min pause for tissue recovery before insertion of the pipette at the next site. Acrylic microbeads were used to confirm accuracy of the microinjection placement based on the Rat Brain Atlas . The Institutional Animal Care and Use Committee and Biosafety Committees of Howard University approved the procedures.

**HSV-1–Based Amplicon Vectors.** HSV-1 amplicons are bacterial plasmids that contain two noncoding elements from HSV- 1, an origin of DNA replication and a DNA packaging/cleavage signal. These elements allow replication and packaging into HSV-1 particles as a 150-kb concatamer. Numerous copies of the transgene sequences are packaged into one vector particle, thereby allowing for high expression levels. The amplicons do not express viral proteins and are not toxic . The construction and properties of the HSV-1 amplicon vector for TLR4 siRNA delivery (pHSVsiTLR4) were previously described . Therapies based on the use of replication-conditional HSV-1 vectors are particularly well suited for the treatment of CNS diseases because amplicons retain the HSV natural ***in vivo*** tropism for neurons, particularly after CNS delivery . Neuronal localization is confirmed by EGFP visualization as shown in Fig. S1.


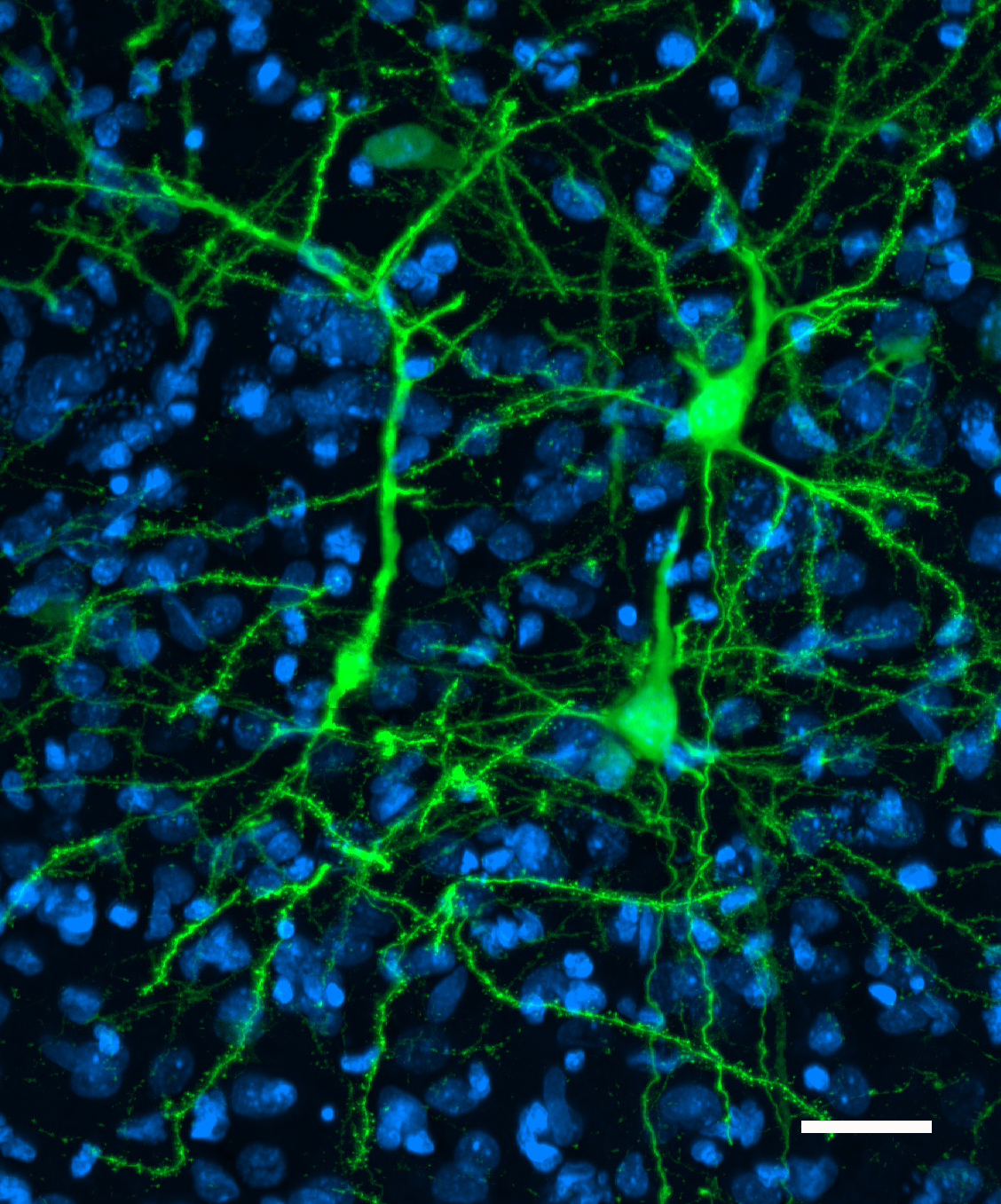


**Fig. S1. Amplicons are neurotropic**. Neurons and neuronal spines and processes located near one of the pHSVsiTLR4 infusion sites show EGFP staining (green). Transduction is effective, with 25–40 neurons at the injection site showing EGFP staining. Glial and other cells stained with DAPI (blue) do not stain for EGFP. Scale bar is 25 µm.

**Small Interfering RNAs and Their Inhibitory Activity.** Small interfering RNAs were designed to target a sequence within the rat TLR4 gene (Gene bank Entry No: NC_005104.2). A scrambled siRNA (NCC) served as control. BLAST search against EST libraries was performed to ensure that no other gene was targeted. The sequences used in these studies are shown in Table S1. They were synthesized as 60-mer sense and antisense oligonucleotide templates (19 × 2 nt) specific to the targeted genes and 22 nt for restriction enzyme sites and hairpin structure). Synthesis was at the University of Maryland Biopolymer Core Facility and used the phosphoramidite (AB) technology. To confirm the ability of the siRNA to inhibit cognate gene expression, RAW 264.7 cells that express TLR4 were transfected with the siRNA at a final concentration of 65 nM using the siPORT amine transfection agent (Ambion) according to the manufacturer’s instructions, and extracts collected 72 h post-transfection were immunoblotted with TLR4 antibody, as previously described .

The pHSVsi vector used to generate the siRNA plasmids that are packaged into HSV-1 virions expresses EGFP under the direction of the HSV-1 IE4/5 immediate-early promoter. The incorporation of EGFP allows for the titration of the vector stocks and the visualization of cell transduction in culture and in the CNS. The pSUPER plasmid, which contains the RNA polymerase III-dependent H1 promoter and well-defined start of transcription and termination signals, is used to generate a second transcription unit for the synthesis of siRNA. The siRNAs were inserted into the pHSVsi vector between the BglII and HindIII sites, downstream of the RNA polymerase III-dependent H1 promoter and packaged as previously described . Briefly, Vero 2–2 cells were transfected with the various plasmids (5 μg) using FuGENE 6 transfection reagent (15 μL) according to the manufacturer’s instructions. After overnight incubation, the cells were infected with 6 × 10^5^ pfu of the HSV-1 5dl1.2 helper virus, and the infected cells were collected 24 h later, when the cytopathic effect was at least 95%. Virus was released by freezing and thawing (virus P1) and further passaged in Vero 2–2 cells to increase the proportion of amplicons relative to helper virus. The P3 virus stock was clarified of cell debris by centrifugation and stored at -80 °C until further use. Virus titers were estimated in PC12 cells by counting the EGFP+ cells 24 h after infection (absorbance 480 nm, emission 507 nm). For example, if 90% of the cells in a well infected with 2.5 μL of virus are green, and if it is assumed that each EGFP+ cell represents one infectious unit, then it can be inferred that the 2.5 μL contained 2.7 × 10^6^ transducing units (TU) (i.e., 90% of 3 × 10^6^ cells plated in each well). Based on these calculations, the titers of our amplicon vectors were 1 × 10^9^ and 2 x 10^8^ TU/ml for pHSVsiTLR4 and pHSVsiNCC, respectively.

**Table S1. siRNA sequences used to construct amplicons**

| siRNA Sequence Target nt |
| --- |
| TLR4 AATGCCAGGATGATGCCTC -9 to 10  NCC GCGGCACACGTAGTAAGTT scrambled |

**SD RESULTS**

**Behavioral Toxicity Measures**. To examine whether amplicons are toxic at the behavioral level, we examined body-weight gain and multiple parameters of locomotor behavior before and following amplicon infusions. Body-weight gains for the amplicon-treated rats were similar to those seen for the PBS control animals. Both groups were observed to nonsignificantly increase their postsurgical weight at 4 d post-treatment. None of the amplicon treatments altered ambulation 4 d post-surgery relative to the PBS control. Thus, open-field behaviors of the two groups were similar. These data, taken along with the failure of the amplicon treatment to alter body-weight measures, suggest that treatment was not associated with overt behavioral toxicity effects and amplicons do not have gross or histologic toxicity related to the type of siRNA, their construction, or the dose and number of intracranial injections. EGFP tracking indicated that the amplicons did not traffic to brain regions distal from the microinjection site. This is consistent with other and previous findings (1,3,5).

**Amplicons Do Not Induce Apoptosis in the CNS Following Intrastriatal Delivery**. Intracranial HSV-1 injection causes fatal encephalitis that is histologically characterized by cell death and apoptosis in 35% to 45% of the cells . Therefore, apoptosis was used as a marker of amplicon-induced relatively rare histologic toxicity effects. Mice were given pHSVsiTLR4, pHSVsiNCC, or PBS (n = 4 each) at 10 sites in the striatum (1.5 × 10^5^ TU each) and followed for 100 d, as previously described . There was no evidence of physical or behavioral toxicity, and striatal sections collected at this time did not stain with antibody to activated caspase-3 (1–3% positive cells in all four study groups). This finding is in contrast to mice (n = 4) given HSV-1 at a 10- fold lower dose (2 × 10 ^4^pfu) and at only one striatal site that died on days 5 to 11 post-injection and had 40% to 55% caspase-3p20+ (activated caspase-3) cells in the striatum . These data indicate that amplicons do not have gross or histologic toxicity related to the type of siRNA, their construction, or the dose and number of intracranial injections. EGFP tracking indicated that the amplicons did not traffic to brain regions distal from the microinjection site.

**The TLR4 and TH levels in the VTA from Wistar rats are similar to those in SD rats and significantly lower than in P rats.** Outbred Wistar rats (n = 10; 3 – 4 months old; 250-550g) were obtained from Harlan Laboratories. To examine whether the levels of TLR4 and TH differ in alcohol-drinking P as compared to Wistar rats, protein extracts from VTA micropunches were immunoblotted with TLR4 antibody and the stripped blots were re-probed with antibodies to TH followed by GAPDH, used as gel loading control. The results were quantitated by densitometric scanning and expressed as densitometric units normalized to GAPDH, as described in MS, Materials and Methods. The levels of both TLR4 and TH in Wistar rats were similar to those seen in SD rats (MS, Fig. 1A) and significantly (p ≤ 0.05) lower than those in P rats (Fig. S2A). Furthermore, double immunofluorescent staining with differentially labeled secondary antibodies (Fig. S2B) confirmed that TLR4/TH co-localization in Wistar rats resembles that seen for SD rats (MS, Fig. 1C), with both lower intensity and % staining cells (10 ± 4.9%) than that seen in P rats (p ≤ 0.05) (MS, Fig. 1B). Collectively, the data confirm that Wistar and SD rats are similar in terms of TLR4/TH expression/cellular localization and significantly different from the P rats.


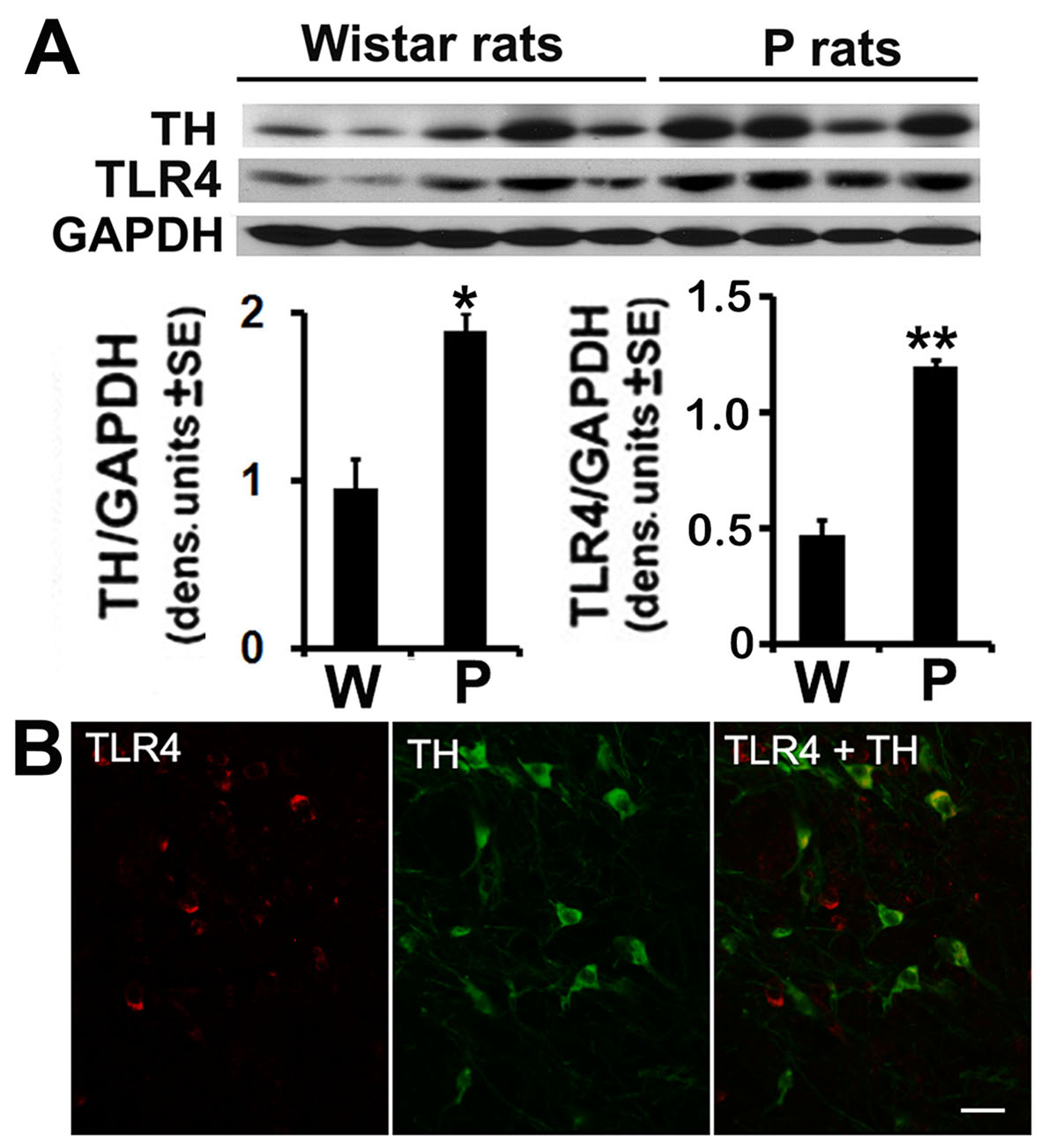


**Fig. S2. Wistar rats have reduced levels of TLR4 and TH in the VTA compared to P rats. (A).** Protein extracts of micropunches collected from the VTA of P (n=5) and Wistar (n=5) rats were immunoblotted with antibodies to TLR4, TH and GAPDH and results are expressed as GAPDH-normalized densitometric units ± SEM. The levels of TH and TLR4 in Wistar rats are similar to those seen for SD rats (MS, Fig. 1A) and significantly lower than those in P Wistar rats (*, p < 0.05; **, p< 0.01 by ANOVA). **(B):** Confocal microscopy and Z-stack imaging of double immunofluorescent staining with TLR4 (red) and TH (green) antibodies is shown for the VTA of Wistar rats. The data are similar to those obtained for the SD rats (MS, Fig. 1C) with significantly lower staining intensity and number of TLR4/TH+ positive cells (10 ± 4.9%) than those seen in the P rats (MS, Fig. 1B). Scale bar: **B:** 20 µm.

**TLR4 is located in GABAergic (GAD1+) neurons in the VTA.** The two major neuronal subpopulations in the VTA are the dopaminergic (TH+) (55%) and GABAergic (GAD1+) (38%) neurons . Having seen that the percentage of dopaminergic neurons that express TLR4 is significantly higher in P than SD rats (61 ± 5.2% and 12 ± 5.5%, respectively) (MS Fig. 1B,C), we wanted to know whether TLR4 is also expressed in GABAergic neurons, with a similar preferential distribution in P as compared to SD rats. Double immunofluorescent staining using TLR4 and GAD1 antibodies, confirmed that TLR4 is also expressed in GABAergic neurons, as shown in Fig. S3 for P rats. TLR4 staining was seen in 83.3% of the GAD1+ cells. Similar co-localization patterns were also seen in the wild type (WT) rats (SD and Wistar), although the intensity of the TLR4 staining was significantly lower, consistent with the lower levels of TLR4 expressed in these animals (MS, Fig. 1C).

**
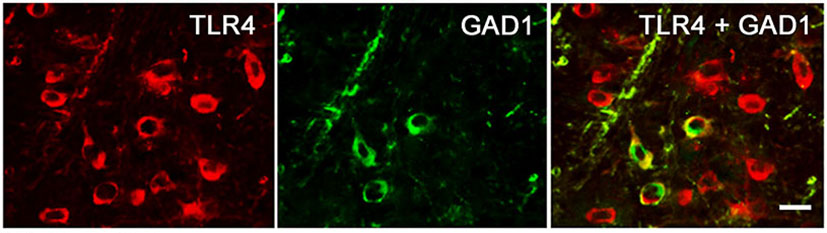
**

**Fig. S3. GABAergic neurons in the VTA express TLR4.** Confocal microscopy and Z-stack imaging of double immunofluorescent staining for TLR4 and GAD1 is shown for the VTA of P rats. Merged images for TLR4 (red) and GAD1 (green) reveal co-expression of TLR4 and GAD1. Scale bar is 20 µm.

**α2 localization in dopaminergic neurons is similar in P and SD rats.** To examine whether TLR4 localization in dopaminergic neurons in P as compared to SD rats, is specific or extends to other genes associated with binge drinking, duplicate sections obtained from P and SD rats were stained in double immunofluorescence with antibodies to TH and α2. We conclude that this is specific for TLR4, because α2 was also co-localized with TH (Fig. S4), but the % cells staining with antibodies to TH and α2 was similar in P and SD rats (86.2 ± 4.0 and 87 ± 3.8%, respectively).


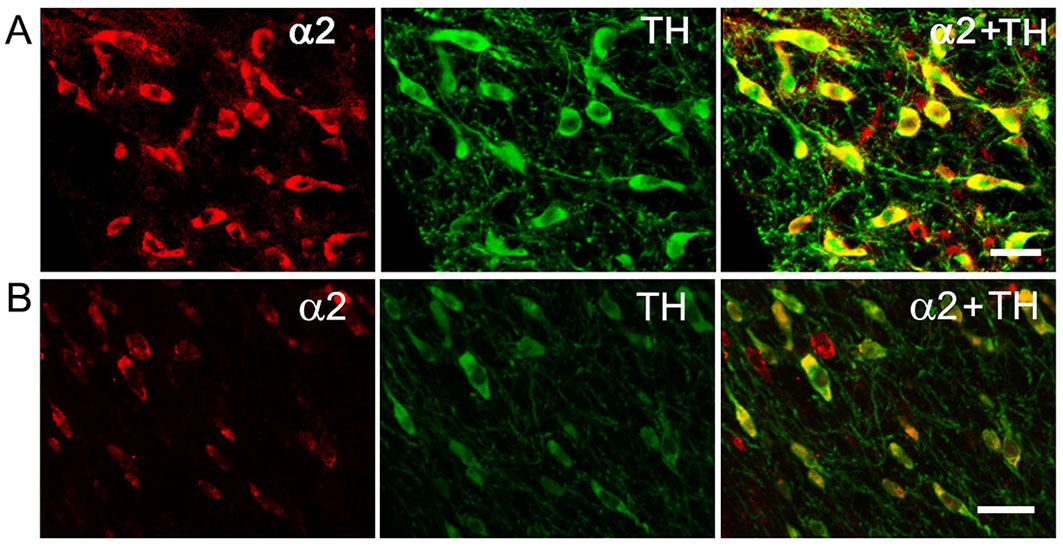


**Fig. S4. TH+ neurons in the VTA of P and SD rats express α2.** Confocal microscopy and Z-stack imaging of double immunofluorescent staining for α2 and TH is shown for the VTA of P **(A)** and SD **(B)** rats. Merged images for α2 (red) and TH (green) reveal numerous TH+ neurons expressing α2 in both P (A) and SD (B) rats**.** Although the staining intensity for both α2 and TH is lower in SD than P rats, the percentage of TH+ cells expressing α2 is similar. Scale bars: **A:** 20 µm; **B:** 25 µm.

**pCREB expression in TH+ neurons is primarily intranuclear**. Having seen that TLR4 induces TH expression through PKA/CREB activation, as also documented by the intranuclear localization of pCREB (MS, Fig. 2B), we wanted to confirm that pCREB is rarely cytosolic in the TH+ neurons in the VTA from P rats. Indeed, double immunofluorescent staining revealed rare TH+ cells with cytosolic pCREB staining (Fig. S5).

**
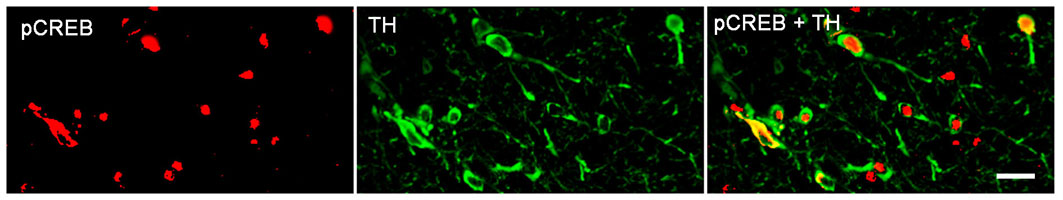
**

**Fig. S5. pCREB cytoplasmic localization in TH+ neurons is rare.** Confocal microscopy and Z-stack imaging of double immunofluorescent staining with pCREB and TH antibodies is shown for the VTA of P rats. Merged images for pCREB (red) and TH (green) reveal that TH+ neurons rarely express cytosolic pCREB. Scale bar is 25 µm.

**TH+ neurons in the VTA from SD rats do not express pPKA/pCREB.** Having seen that pPKA/pCREB are expressed in TLR4+ dopaminergic (TH+) neurons from the VTA of P rats (MS, Fig. 3), we wanted to know whether this is also true in SD rats. Double immunofluorescent staining of sections collected from the SD rats indicated that TLR4 is barely expressed in these animals (Fig. S6B) and this correlates with the absence of pPKA and pCREB expression (Fig. S6). Staining patterns in Wistar rats were similar to those seen for the SD rats.

**
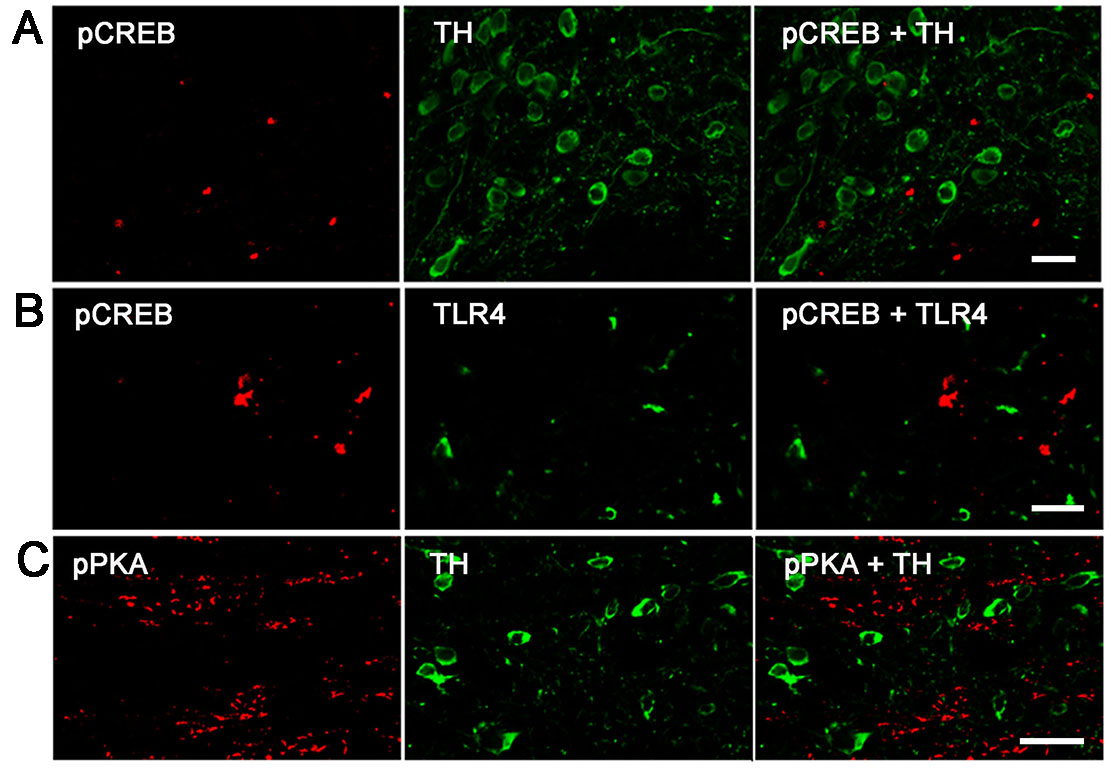
**

**Fig. S6. TH+ neurons do not express pCREB and pPKA in the VTA of SD rats.** Confocal microscopy and Z-stack imaging of double immunofluorescent staining for pCREB/TH, pCREB/TLR4, and pPKA/TH is shown for the VTA of SD rats. Merged images for pCREB (red) and TH (green), pPKA (red) and TH (green) reveal no co-expression of TH and pCREB or TH and pPKA in the VTA of SD rats and randomly scattered TLR4+ structures do not co-express pCREB. Scale bars: A: 30 µm; B: 40 µm; C: 55 µm**.**

**REFERENCES**

1. Liu J, Yang AR, Kelly T, Puche A, Esoga C, June HL, Jr., et al. Binge alcohol drinking is associated with GABAA alpha2-regulated Toll-like receptor 4 (TLR4) expression in the central amygdala. Proceedings of the National Academy of Sciences of the United States of America. 2011;108(11):4465-70. Epub 2011/03/04.

2. Paxinos G, Watson C. The Rat Brain in Stereotaxic Coordinates. 6 ed. San Diego, CA: Academic Press; 2009.

3. June HL, Liu J, Warnock KT, Bell KA, Balan I, Bollino D, et al. CRF-amplified neuronal TLR4/MCP-1 signaling regulates alcohol self-administration. Neuropsychopharmacology : official publication of the American College of Neuropsychopharmacology. 2015;40(6):1549-59. Epub 2015/01/09.

4. Harvey SC, Foster KL, McKay PF, Carroll MR, Seyoum R, Woods JE, 2nd, et al. The GABA(A) receptor alpha1 subtype in the ventral pallidum regulates alcohol-seeking behaviors. The Journal of neuroscience : the official journal of the Society for Neuroscience. 2002;22(9):3765-75. Epub 2002/04/30.

5. Saydam O, Glauser DL, Heid I, Turkeri G, Hilbe M, Jacobs AH, et al. Herpes simplex virus 1 amplicon vector-mediated siRNA targeting epidermal growth factor receptor inhibits growth of human glioma cells in vivo. Molecular therapy : the journal of the American Society of Gene Therapy. 2005;12(5):803-12. Epub 2005/08/23.

6. Perkins D, Gyure KA, Pereira EF, Aurelian L. Herpes simplex virus type 1-induced encephalitis has an apoptotic component associated with activation of c-Jun N-terminal kinase. Journal of neurovirology. 2003;9(1):101-11. Epub 2003/02/15.

7. Taylor SW, Smith RM, Pari G, Wobeser W, Rossiter JP, Jackson AC. Herpes simplex encephalitis. The Canadian journal of neurological sciences Le journal canadien des sciences neurologiques. 2005;32(2):246-7. Epub 2005/07/16.

8. Saeki Y. Stable CNS gene delivery with Sleeping Beauty armed with a high-capacity HSV virion. Molecular therapy : the journal of the American Society of Gene Therapy. 2006;13(3):457-8. Epub 2006/02/08.

9. Berges BK, Wolfe JH, Fraser NW. Transduction of brain by herpes simplex virus vectors. Molecular therapy : the journal of the American Society of Gene Therapy. 2007;15(1):20-9. Epub 2006/12/14.

10. Suzuki M, Chiocca EA, Saeki Y. Stable transgene expression from HSV amplicon vectors in the brain: potential involvement of immunoregulatory signals. Molecular therapy : the journal of the American Society of Gene Therapy. 2008;16(10):1727-36. Epub 2008/08/30.

11. Manservigi R, Argnani R, Marconi P. HSV Recombinant Vectors for Gene Therapy. The open virology journal. 2010;4:123-56. Epub 2010/09/14.

12. Cohen M, Braun E, Tsalenchuck Y, Panet A, Steiner I. Restrictions that control herpes simplex virus type 1 infection in mouse brain ex vivo. The Journal of general virology. 2011;92(Pt 10):2383-93. Epub 2011/06/24.

13. de Silva S, Bowers WJ. Targeting the central nervous system with herpes simplex virus / Sleeping Beauty hybrid amplicon vectors. Current gene therapy. 2011;11(5):332-40. Epub 2011/06/30.

14. Fiandaca MS, Bankiewicz KS, Federoff HJ. Gene therapy for the treatment of Parkinson's disease: the nature of the biologics expands the future indications. Pharmaceuticals. 2012;5(6):553-90. Epub 2012/01/01.

15. Aurelian L. Herpes Simplex Viruses: General Features. In: Mahy BWJ, van Regenmortel MHV, editors. Encyclopedia of Virology. 3 ed: Elsevier, ltd; 2014. p. 383–97.

16. Smith CC, Peng T, Kulka M, Aurelian L. The PK domain of the large subunit of herpes simplex virus type 2 ribonucleotide reductase (ICP10) is required for immediate-early gene expression and virus growth. Journal of virology. 1998;72(11):9131-41. Epub 1998/10/10.

17. Wales SQ, Li B, Laing JM, Aurelian L. The herpes simplex virus type 2 gene ICP10PK protects from apoptosis caused by nerve growth factor deprivation through inhibition of caspase-3 activation and XIAP up-regulation. Journal of neurochemistry. 2007;103(1):365-79. Epub 2007/09/20.

18. Laing JM, Golembewski EK, Wales SQ, Liu J, Jafri MS, Yarowsky PJ, et al. Growth-compromised HSV-2 vector Delta RR protects from N-methyl-D-aspartate-induced neuronal degeneration through redundant activation of the MEK/ERK and PI3-K/Akt survival pathways, either one of which overrides apoptotic cascades. Journal of neuroscience research. 2008;86(2):378-91. Epub 2007/09/26.

19. Yang AR, Liu J, Yi HS, Warnock KT, Wang M, June HL, Jr., et al. Binge Drinking: In Search of its Molecular Target via the GABA(A) Receptor. Frontiers in neuroscience. 2011;5:123. Epub 2011/10/25.

20. Golembewski EK, Wales SQ, Aurelian L, Yarowsky PJ. The HSV-2 protein ICP10PK prevents neuronal apoptosis and loss of function in an in vivo model of neurodegeneration associated with glutamate excitotoxicity. Experimental neurology. 2007;203(2):381-93. Epub 2006/10/19.

21. Nair-Roberts RG, Chatelain-Badie SD, Benson E, White-Cooper H, Bolam JP, Ungless MA. Stereological estimates of dopaminergic, GABAergic and glutamatergic neurons in the ventral tegmental area, substantia nigra and retrorubral field in the rat. Neuroscience. 2008;152(4):1024-31. Epub 2008/03/22.
